# Disorders of the Pleural Space: Gas, Liquid, and Solid

**DOI:** 10.1155/2012/594286

**Published:** 2012-06-19

**Authors:** Joseph S. Friedberg, Takashi Nakano

**Affiliations:** ^1^Division of Thoracic Surgery, Department of Surgery, University of Pennsylvania, Philadelphia, PA 19104, USA; ^2^Division of Respiratory Medicine, Department of Internal Medicine, Hyogo College of Medicine, Nishinomiya, Hyogo 19104, Japan

This special issue focuses on the pleural space, a unique region of the human body affected by some of the earliest described maladies in medical science yet remaining a mystery in both purpose and function. The two pleural spaces, defined by the bony thorax, diaphragm, and mediastinum, are each occupied by the lungs. Consequently, the pathophysiologic disorders of the space involve not only benign and malignant disorders that take the form of solid masses or effusions, but also gaseous disorders as well. 

Ongoing air leakage through the surface of a lung will result in collapse and, if ongoing, will result in a fatal pneumothorax. These are some of the most common problems encountered in the pleural space and are treated, along with other maladies, with tube access of the pleural space. Although a common procedure, it requires technical precision and judgment to avoid potentially disastrous complications, as detailed in this special issue. Embryologic development of the pleural structures, discussed in this special issue, results in planes of dissection and paths of least resistance that can result in unusual and unexpected patterns of air accumulation when the air leaks within the lungs. 

The net negative pressure within the pleural space and the positive pressure in the pulmonary arterial circulation and the lymphatic circulation maintain a dynamic equilibrium in pleural space, the net effect being a minimal amount of fluid at any given time despite a high flow from the visceral to parietal pleural surfaces. Perturbation of this delicate balance results in fluid accumulation. Because of the extraordinary number of factors involved in maintaining this balance, it can be very difficult to diagnose the reason for the fluid buildup. Within the context of the overall clinical picture, the diagnosis is often rendered by analysis of the fluid itself. As described in this special issue, there are many tests and criteria accessible to the clinician to diagnose the etiology of the fluid accumulation. When the fluid is caused by a malignancy or noninfectious benign process, it is typically necessary to intervene and stop the fluid accumulation. The most common approach remains pleurodesis, affecting a symphysis between the visceral and parietal pleural surfaces for the purpose of obliterating the space. Using talc for this purpose remains one of the most common, and arguably best, techniques and is described in this special issue.

When the fluid does accumulate and becomes infected, it is called an empyema, and this too can easily escalate into a fatal condition without prompt and appropriate treatment. Although drainage, as first described by Hippocrates over 2000 years ago, remains a critical element of the treatment, there are now multiple tools and techniques available to the clinician to combat this common and lethal disorder. A review of this important topic is covered in this special issue. Although the pleural space is highly resistant and resilient in the face of infectious challenges, the ultimate clearance and recovery most often hinges on full lung expansion with pleural-pleural apposition. When an empyema is not drained early enough, the lung can become encased in a fibrous peel that precludes reexpansion and results in a chronic space. Ideally, the fibrous peel can be surgically resected to reexpand the lung and fill the space. At times, however, the lung will no longer fill space, and this results in a challenging and deadly situation that warrants surgical intervention. As described in this special issue, this can take the form of opening the space to allow full drainage to obliterating the space by filling it with healthy vascularized tissue and/or collapsing the chest wall to meet the lung surface.

The pleural space is a fascinating and enigmatic region of the body maintained in balance by a symphony of homeostatic mechanisms. Perturbation of this balance by benign or malignant processes can lead to a myriad of problems, unique to the pleural space. Addressing these problems requires an understanding of the embryology, anatomy, and physiology and then combining this knowledge with sound judgment, scientific analysis, meticulous technique, and, at times, a dash of detective work. We hope you enjoy this special issue as it explores these topics. 

*Joseph S. Friedberg* *Joseph S. Friedberg* *Takashi Nakano* *Takashi Nakano*

